# Lameness in fattening pigs – *Mycoplasma hyosynoviae*, osteochondropathy and reduced dietary phosphorus level as three influencing factors: a case report

**DOI:** 10.1186/s40813-020-00184-w

**Published:** 2020-12-15

**Authors:** B. Wegner, J. Tenhündfeld, J. Vogels, M. Beumer, J. Kamphues, F. Hansmann, H. Rieger, E. grosse Beilage, I. Hennig-Pauka

**Affiliations:** 1 Veterinary Practice Duemmerland, Steinfeld, Oldenburg, Germany; 2 Vetland® Dr. Tenhündfeld & Kollegen, Vreden, Germany; 3 Field Station for Epidemiology, University of Veterinary Medicine Hannover, Foundation, Hannover, Germany; 4 Institute for Animal Nutrition, University of Veterinary Medicine Hannover, Foundation, Hannover, Germany; 5 Department of Pathology, University of Veterinary Medicine Hannover, Foundation, Hannover, Germany

**Keywords:** Locomotor disorder, Mineral supply, *Mycoplasma hyosynoviae*, Nutrition, Swine

## Abstract

**Background:**

Multiple diagnostic procedures, their results and interpretation in a case with severe lameness in fattening pigs are described. It is shown that selected diagnostic steps lead to identification of various risk factors for disease development in the affected herd. One focus of this case report is the prioritization of diagnostic steps to verify the impact of the different conditions, which finally led to the clinical disorder. Assessing a sufficient dietary phosphorus (P) supply and its impact on disease development proved most difficult. The diagnostic approach based on estimated calculation of phosphorus intake is presented in detail.

**Case presentation:**

On a farrow-to-finishing farm, lameness occurred in pigs with 30–70 kg body weight. Necropsy of three diseased pigs revealed claw lesions and alterations at the knee and elbow joints. Histologic findings were characteristic of osteochondrosis. All pigs were positively tested for *Mycoplasma hyosynoviae* in affected joints. *P* values in blood did not indicate a P deficiency, while bone ashing in one of three animals resulted in a level indicating an insufficient mineral supply. Analysis of diet composition revealed a low phosphorus content in two diets, which might have led to a marginal P supply in individuals with high average daily gains with respect to development of bone mass and connective tissue prior to presentation of affected animals. Finally, the impact of dietary factors for disease development could not be evidenced in all submitted animals in this case.

**Conclusions:**

*Mycoplasma* (*M*.) *hyosynoviae* was identified to be an important etiologic factor for disease. Other, non-infectious factors, such as osteochondrosis and claw lesions might have favored development of lameness. In addition, a relevant marginal P supply for pigs was found in a limited time period in a phase of intense growing, but the potential interaction with infection by *M. hyosynoviae* is unknown.

The presented case of severe lameness in fattening pigs revealed that three different influences presumably act in pathogenesis. Focusing only on one factor and ignoring others might be misleading regarding subsequent decision-making for prevention and therapy. Finally, clinical symptoms disappeared after some changes in diet composition and anti-inflammatory treatment of individual animals.

## Background

*Mycoplasma* (*M*.) *hyosynoviae* is a commensal of the upper respiratory tract, especially in the tonsils [1], that may lead to clinical disease mainly in older pigs (> 10 weeks) [2]. The detection rate has increased in the last years in pigs with arthritis in Denmark with approximately 20% already in 2001 [3], and in the USA with approximately 8% in 2003, 23% in 2010 [2] and 26% in 2015 [4]. Affected pigs show avoidance of standing up or an impaired ability to stand within approximately 24 h post infection [5, 6]. The clinical symptoms include sudden lameness affecting one or more limbs, with the animals balancing their weight from one leg to the other [6] up to a dog sitting position [7]. Young adults often experience two-three lameness episodes over a 4-6-week period [8]. *M. hyosynoviae* is probably present in many herds worldwide, high morbidity rates occurring in fattening pigs between 50 and 60 kg; gilts and breeding boars could be affected as well [1]. It is also known that joint infections with *M. hyosynoviae* can be clinically asymptomatic, since the pathogen has also been diagnosed in synovial fluid of non-lame pigs [3]. For this reason, additional trigger factors for disease development should be taken into account during the diagnostic procedure.

A common degenerative disorder in skeletally immature pigs is osteochondrosis, which is initiated by failure of the blood supply in the growth plate cartilage and ischemic chondronecrosis resulting also in bone lesions [9–11]. The etiology of osteochondrosis is mostly multifactorial, with several factors triggering disease development as heritable and anatomical traits and physical trauma, while rapid growth and dietary factors were also discussed [12]. Specific anatomical traits such as unfavorable joint shapes, carcass length and weight of hams were correlated with osteochondrotic lesions, especially if combined with biomechanical stressors [13, 14].

Other important diseases in growing pigs are metabolic systemic osteopathies due to mineral imbalances. In recent years, the German Fertilizer Ordinance (FO) has been made stricter, with the aim of reducing the environmental nitrogen and phosphorus load. According to national and international legislation, several restrictions in manuring came into force to reduce emissions from intensive animal production [15–17]. A high potential measure for emission reduction is an N- and P-reduced feeding concept strictly adapted to the animal’s requirements [18]. Reference standard concentrations for nutrients in animal feedstuff are commonly used for calculating the necessary amount of nutrients in a diet to fulfil animal needs. Usually it is not taken into account that dietary nutrient levels vary depending on different growth and weather conditions. For this reason, the routinely performed conception of a diet, which is based on reference standard concentrations, bears the risk of a marginal nutrient supply. A specific problem is the correct quantification of digestible P, which cannot be defined by chemical analysis of the diet, but by feeding experiments only. The digestible P levels vary between but also within different crops, especially due to phytate as well as phytase levels [19].

Phosphorus, in particular, is frequently reduced in the diet of pigs to lower P-content in manure and to save costs for this expensive component. General weight-group dependent estimates for digestible P in diets of 3.3 g/kg (20–28 kg body weight (bw)) and 3.1 g/kg (20–40 kg bw) are oriented according to growth demands [20]. Digestible P is primarily used for growth and is in parallel accumulated in bones with increasing concentrations until a plateau is reached and excess P is excreted also in urine [21]. In general, requirements for digestible P are higher for bone mineralization than for maximizing gains and are defined to be approximately 4.3–5.5 g/kg [21–23] for pigs with 20–40 kg bw, which were also dependent on the Ca: digestible P ratio, which should be approximately 2.5:1 [22].

Reduced dietary P levels in the weaner-grower period can lead to impaired bone mineralization [24]. It has to be underlined that bone mineralization in the early growth phase of mammals is fundamental for maximum bone mineralization in later life, and is therefore crucial for prevention of locomotor disorders [25]. P utilization is not only dependent on the form of the P sources used (inorganic vs. organic), but also on the activity of phytases present in the diet (endogenous or added) and the Ca:P ratio [26]. Both elements are tightly regulated in parallel to maintaining homeostasis with bone as a target but also as endocrine tissue for the regulating hormones and modulating factors [27].

A minimum Ca:P ratio of 1:1 is required, while the recommendation for pigs with a 50–80 kg bw is 1.25:1 [20]. Dietary Ca concentrations for maximal mineralization were highly variable between different studies (6.5–10 g/kg) [22]. A Ca deficiency can lead to a weakening of the growth plate located between meta- and epiphysis at the ends of long bones, resulting in development of osteoid as an unmineralized organic bone matrix [28, 29]. Other nutritional factors, such as vitamin D supplementation, influence the Ca-P homeostasis [30]. Adequate supply of minerals for bone development might also be dependent on the general herd health because metabolic changes during infection and inflammation, immune deficiency or disease-related stress can alter feed uptake and protein accretion [31]. In addition, age, gender, economic and several biological factors influence bone development [32].

**Fig. 1:**
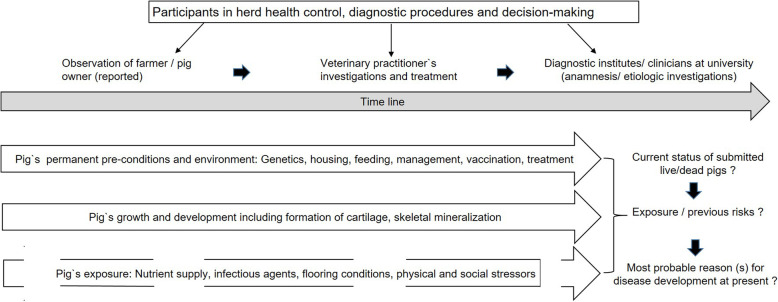
Schematic timeline indicating common challenges for veterinary diagnostic activities in case of later involvement and faced with a herd health problem with a case history of unknown duration

In the present case report, it became apparent that the veterinary practitioner and the involved diagnostic institute faced a disease picture, which represented the final outcome of the exposure to etiologic causes dating back to earlier periods of life-time (Fig. 1). It was shown that three different etiologic factors were interlocked during pathogenesis of lameness in fattening pigs. The report describes a large panel of diagnostic measures. Finally, the most straightforward diagnostic approach covering the different facets of “lameness” is discussed as a lesson for clinical practice learnt from this case.

## Case history

On a farrow-to-finishing farm with 450 productive sows and 4900 fattening pigs in North Rhine-Westphalia, Germany, lameness occurred in fattening pigs of both sexes with a body weight of 40–70 kg. Male pigs were not castrated on this farm. The genetic background of sows was DanBred, while the piglets were crossbreds of DanBred x PIC 408. The farm produced in a three-week batch system with seven batches in sizes of approximately 64 productive sows and 2.35 litters per sow per year. After a lactation of 4 weeks, piglets were raised in groups of 42 animals per pen up to a weight of 28 kg in the nursery unit on plastic slatted flooring until week 11 of age. The totally slatted flooring in the fattening stable complied with the German Animal Welfare Livestock Farming Regulation with 18 mm gaps and 80 mm wide concrete elements. The slatted plastic flooring in the nursery was assessed as soft ground, while the slatted concrete flooring in the fattening stable was assessed as hard. In total, the nursery had approximately 3000 places and the fattening stable approximately 4900 places with 35 pigs per pen. All-in-all-out (AIAO) was performed in all production units. Average daily weight gain was 820 g in fatteners within an average fattening period of 110 days. The average body weight of pigs at slaughter was approximately 116–118 kg.

The final diet in the trough was a combination of fermented ingredients (cereals and rapeseed meal with 0.14% Ca and 0.4% P) and a supplementary feed (Table 1: AZ-3, VM28, AM40, MM65) in varying proportions for different age groups. The mixture of ingredients that were used in the controlled fermentation (13 h at 37–39 °C) and the supplementary feed were delivered by a conventional feed company. Fermentation resulted in a final pH of 3.8 using starting cultures of *Lactobacillus plantarum* and *Pediococcus acidilactici* (Proferm HCL-FL®, Agravis Raiffaisen AG, Münster, Germany). The fermented part had been automatically added to the final liquid diet immediately before feeding starting at day 15 after weaning. The share of fermented ingredients within the final diet increased from 5% up to 65% in the final fattening period. The final diet contained thermostable and pH-tolerant phytase-6 (4a24 DuPont Axtra®-Phy-thermostabil, DuPont Nutrition & Biosciences, Copenhagen, Denmark). The drinking water was farm own ground water. Samples of the different final liquid diets were analysed by the LUFA/Chamber of Agriculture North Rhine-Westphalia, Germany for nutrient contents (Table 1). Diets were analyzed by standard procedures following the routine methods of the Association of German Agricultural Investigation and Research Institutions (Verband Deutscher Landwirtschaftlicher Untersuchungs- und Forschungsanstalten). Crude protein was determined on the basis of nitrogen quantification following the method of Kjeldahl. Nitrogen was measured in a high temperature elemental analyzer (Vario Max®, Elementar, Hanau, Germany). The calcium content was determined by atomic absorption spectrometry, phosphorus colorimetrically [33, 34]. For the analysis of crude fiber the fat- and ash-free samples were cooked with acid (1.25% sulphuric acid) and alkaline (1.25% sodium hydroxide) solutions in a crude fibre determination device (Fibertec™ 2010 Hot Extractor, Fa. Foss GmbH, Hamburg) followed by drying, ashing and weighing [33].

**Table 1:** Feeding techniques and diets’ composition (in all groups: liquid diet offered ad libitum, probe system)

	Diet (AZ-3) for growing pigs (88% DM)	Diet 1 (VM28) for fattening pigs (88% DM)	Diet 2 (AM40) for fattening pigs (88% DM)	Diet 3 (MM65) for fattening pigs (88% DM)
Week	9–11	12–14	15–17	18–20
Body weight (kg)	15–28	28–40	41–65	66–80
Pigs, n per group	42	35		
Pigs, n per valve	84	70		
Length of trough (m)	2.5	3.8		
**Dry matter** (DM) of the final liquid diet (%)^a^	25.60	21.80	24.40	23.80
**Analyzed nutrients** (88% DM):				
Crude protein (%)	18.10	18.20	16.80	17.40
Crude fiber (%)	3.00	2.90	3.50	3.80
Ca (%)	0.92	0.81	0.87	0.79
P (%)	**0.46**	**0.46**	**0.46**	0.49
Ca:P ratio	2:1	1.8:1	1.9:1	1.6:1
**Labeled nutrient content in supplementary feed** (88% DM):				
Energy (MJ ME)	13.20	13.20	13.20	13.10
Crude protein (%)	18.50	16.14	15.25	14.00
Lysine (%)	1.50	1.15	1.08	0.98
Fat (%)	5.00	3.25	3.00	3.00
Fiber (%)	3.50	4.10	4.25	4.50
Ca (%)	1.00	0.70	0.67	0.65
P (%)	0.45	0.40	0.40	0.37
6-Phytase (FTU/kg)	1125	750	750	750

Analyzed values of diet composition and feeding techniques in the different age groups are shown. P-content is highlighted in bold as it is assessed to be critically low compared with the officially recommended values [20] regarding total dietary phosphorus in Table 2

^a^Share of fermented ingredients varied between 28 and 50%

As shown in the feed declaration in Table 1, compared to official recommendations shown in Table 2, the total phosphorus content was reduced in the diet and did not meet the officially recommended values.

**Table 2:** Official recommendations for feed intake, energy, protein and Ca and P contents according to the weight range of pigs [20]

**Body weight range** (kg)	11–25	25–50	50–75	75–100
**Daily feed intake** (g, 88% DM)	953	1582	2229	2636
**Energy** (MJ ME/kg, 88% DM)	14.0	13.8	13.8	13.8
**Protein** (g/kg, 88% DM)	189	157	138	121
**Calcium** (g/kg, 88% DM)	7.0	6.6	5.9	5.2
**Phosphorus** (g/kg, 88% DM)				
-total	**6.0**	**5.6**	**5.2**	4.7
-digestible	3.3	3.1	2.7	2.4

Recurring diseases on farm were arthritis and meningitis caused by *Streptococcus* (*S*.) *suis*, which was treated by amoxicillin application via the liquid diet in cases of disease outbreaks. Intermittent cases of fever and respiratory distress occurred in fatteners due to the influenza virus. At the time of this case report, neither *S.-suis*- nor influenza-related disease cases were observed. Routinely, all sows were vaccinated against four influenza virus subtypes at day 80 of gestation (Respiporc FLU3 and Respiporc FLUPAN H1N1, CEVA Tiergesundheit GmbH, Düsseldorf, Germany) and against parvovirus and swine erysipelas at day 14 after farrowing (Parvoruvac, CEVA Tiergesundheit, Düsseldorf, Germany). Routinely, all piglets were vaccinated with commercial products against the Porcine Reproductive and Respiratory Syndrome virus (Unistrain® PRRS, Hipra, Amer, Spain), Porcine Circovirus 2 (Ingelvac Circoflex®, Boehringer Ingelheim, Vetmedica GmbH, Ingelheim, Germany) and *M. hyopneumoniae* (Hyogen®, CEVA Tiergesundheit GmbH, Düsseldorf, Germany) at an age of 24 days, i.e., prior to weaning. At an age of 50 and 70 days, pigs were vaccinated against *Actinobacillus pleuropneumoniae* (Coglapix®, CEVA Tiergesundheit GmbH, Düsseldorf, Germany).

Clinical examination of pigs in the pens was performed daily by the farmer by inspecting groups of pigs in pens and counting those pigs with suspected lameness according to behavior during resting, standing up, lying down and walking. Diseased pigs were color-marked on the back for later inspection, treatment and follow-up assessment on the following days. After assessing the number of affected pigs, selected individual pigs were examined by visual examination of the joints by the veterinarian at its weekly visits. Main clinical symptoms were high-grade lameness with a stiff walk in approximately 10–35% of the fattening pigs at an age of 80–140 days (30–70 kg bw). Limbs were free of swellings and no obvious signs of arthritis were observed. All diseased animals and the individual treatments were recorded by the farmer. Treatment data were digitalized within the official antibiotic database of the obligatory German surveillance system. For treating those pigs with lameness, amoxicillin trihydrate (Hostamox LA 150 mg/mL, MSD Tiergesundheit Deutschland GmbH, Unterschleißheim, Germany, 15 mg/kg bw) was injected two-four times at 24 h intervals. In addition, 0.1 mg dexamethasone per kg bw (Rapidexon Albrecht, Dechra Veterinary Products Deutschland GmbH, Aulendorf, Germany) was injected twice with an interval of 1 day. Approximately 90% of the treated pigs recovered within 1 week after treatment. The oral treatment with 10 mg tiamulin fumarate (Denagard 45%, Elanco Deutschland GmbH, Bad Homburg, Germany) per kg bw via the liquid diet was not successful.

## Case presentation

**Fig. 2:**
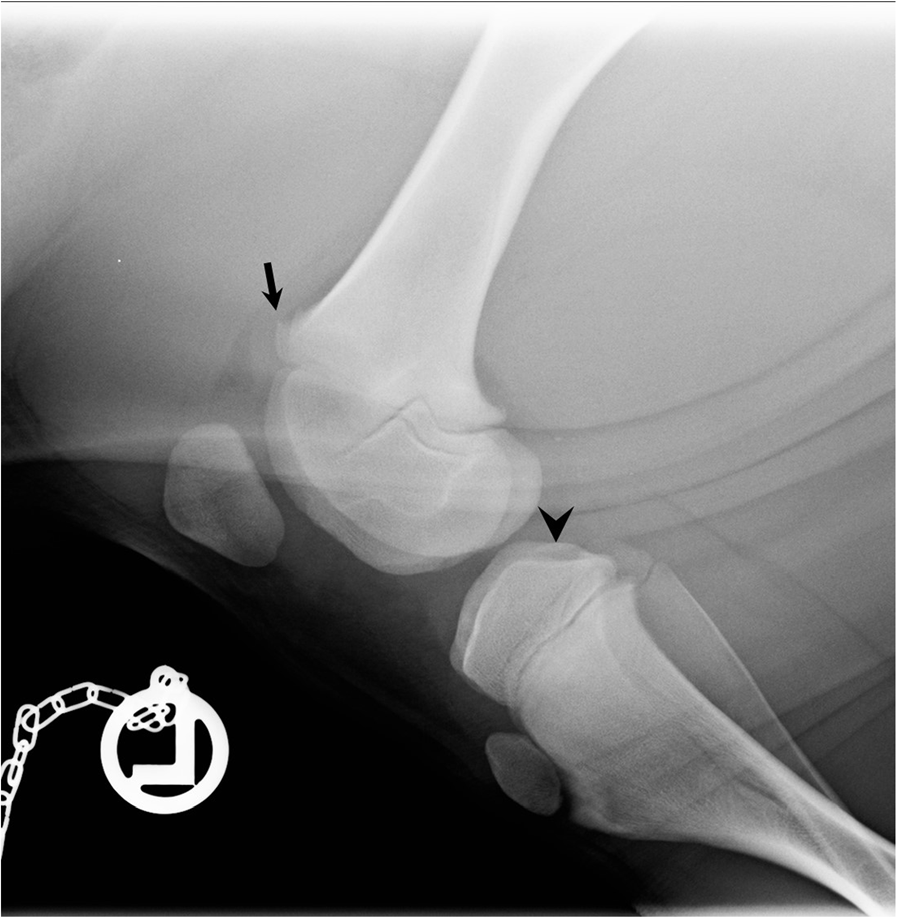
X-ray findings of left knee of pig 3. An osseous tulip-shaped bulge at the distal epiphyseal cartilage of the femur (arrow) as well as incongruities at the articular surface of the tibia (arrowhead) are visible. The x-ray picture was produced with pre-settings 30 kV, 50 kV, 15 mAS

**Fig. 3:**
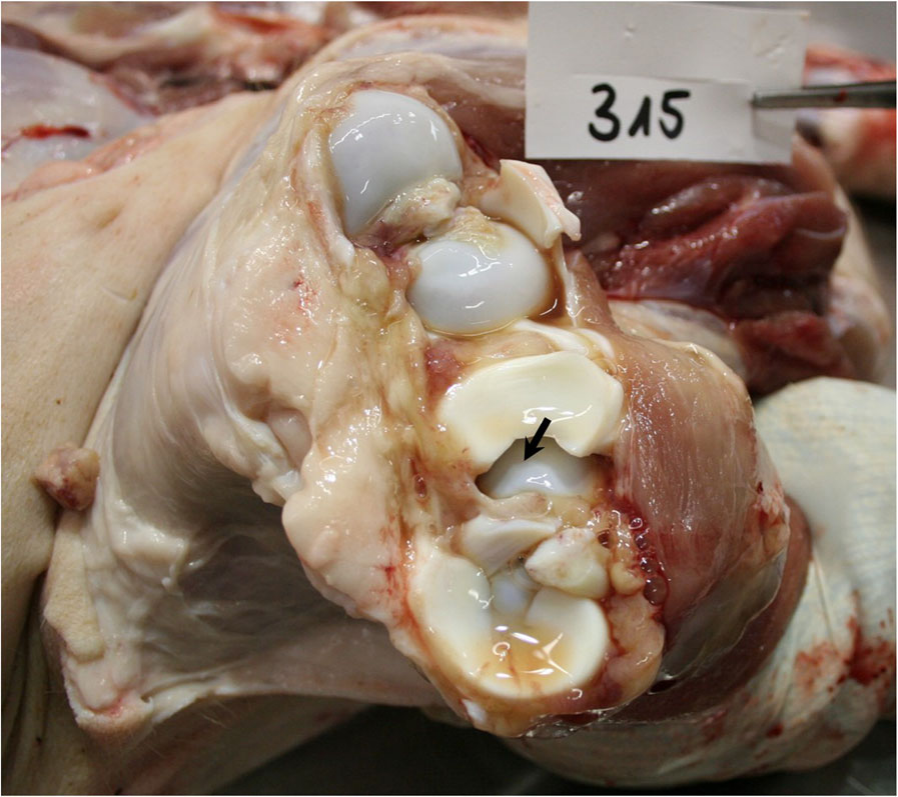
Macroscopic lesions in the knee of pig 3. Irregularities at the articular cartilage of the tibia are visible (arrow)

**Fig. 4:**
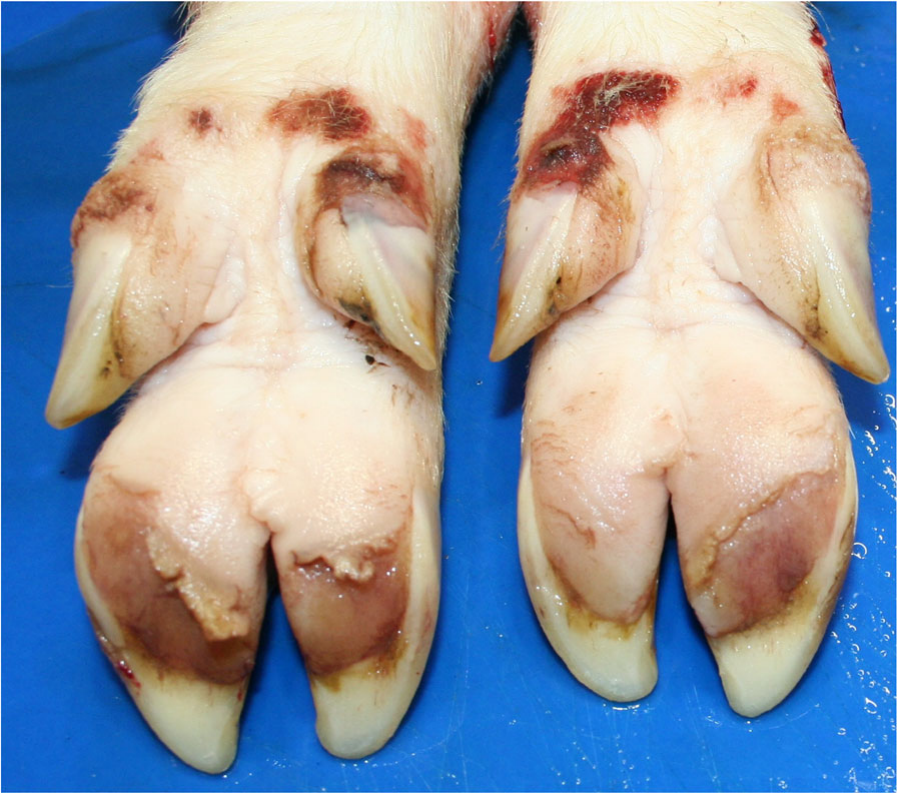
Macroscopic lesions at claws of pig 1. Volar surface of claws of the hind feet with erosive heel lesions and lesions of the coronary band at the dew claws. In this pig, erosive heel lesions were found in all claws

In February 2019, three untreated pigs (32–45 kg bw) from the affected age-groups showing severe tripping in the hind legs were selected by the veterinarian for further diagnostics. The attending veterinarian performed X-rays of limbs under deep anesthesia after intramuscularly administering 2 mg azaperone/kg bw (Stresnil®, Elanco Deutschland GmbH, Bad Homburg, Germany) and 20 mg ketamine/kg bw (Ursotamin®, Serumwerk Bernburg, Bernburg, Germany). Only those joints with high probability of osteochondrosis based on observed movement behavior of the animals (tripping in hind limbs) were x-rayed. Joints selected for x-raying are described in Table 3. Pigs 1 and 3 were reluctant to move the forelimbs before standing up, so that the shoulder and elbow joints were x-rayed as well. Digital x-ray examination of the joints was performed in a radiological unit (Vetsystem 30, IBM Inc., Armonk, NY, USA) with automatic exposure with 30 kW. Detailed pre-setting values are recorded in Table 3. Anesthetized animals were placed in lateral or sternal recumbency depending on the x-ray path. Slight irregularities were found in the epiphyseal transition zone at the knees of pig 2 and pig 3 (Fig. 2) as well as the elbow joints in pig 1. No other joints showed pathologic findings in x-ray pictures. Subsequently, pigs were transported to the Field Station for Epidemiology of the University of Veterinary Medicine Hannover in Bakum, Germany, where a further clinical examination was performed prior to euthanasia. Examination of the limbs following good veterinary practice, starting with inspection from behind, from the front and the side during standing and walking of the animal, was performed prior to euthanasia. The character (during support or hanging of the limb) and severity grade of lameness, as well as the affected anatomical side was assessed by inspection, followed by palpation of the joints. Lameness was characterized by stiff walking and a quick change in weight-strain between the hind limbs during standing (tripping). All animals were reluctant to stand up. No obvious joint swellings were observed. Blood samples were taken from the *V. jugularis* of these pigs prior to euthanasia. Blood was centrifuged after a clotting time of approximately 30 min at 2000 g for 10 minutes. Pre-analytical treatment of blood samples is critical for P analysis because hemolysis leads to an artificial increase in P concentrations. For this reason, hemoglobin was measured in the serum samples as a quality control. Macroscopic necropsy findings are summarized in Table 3. Macroscopic cartilage lesions are shown in Fig. 3 and claw lesions in Fig. 4. Cultural microbiological testing of articular swabs showed no pathogens. Real-time PCR was positive for *M. hyosynoviae* (cycle threshold (ct) value 29–32) in all three animals.

**Table 3:** Macroscopical findings regarding joints and claws of the three pigs

	Pig 1 (female, 45 kg)	Pig 2 (male, 32 kg)	Pig 3 (female, 39 kg)
**Joints**	Elbow and tarsal joint: Slightly increased amount of synovia, turbid synovia, redness of synovialis.	Carpal joint: Subcutaneous edema of the joint. Knee joint: Slight increase in synovia, turbid synovia.	Carpal joint: Slightly increased amount of synovia, turbid synovia, redness of synovialis. Tarsal joint: Subcutaneous edema of the joint.
**Claws**	Both claws of both forelimbs: Medial dew claws: 2 × 1 cm^2^ lesion at the lateral wall. All dew claws: skin of the coronary band not intact. Left claw of hindlimb: Lateral claw and dew claw each: 1 × 1 cm^2^ lesion at lateral wall. All claws: Superficial erosive heel lesions (horn detachment at the heel sole horn).	Lateral claw of left forelimb: Upper sole layer lost, lower layers appear dark and rough. All claws: Superficial horn detachments in the cranial parts of the heel sole horn.	Lateral claws of both forelimbs: Wall horn fissures in the caudal part with slight wall horn detachment from corium in an area of 0.5 × 1 cm^2^.
**X-ray pictures**	Hips (66 kV, 30 mAs), knees (50 kV, 20 mAs), shoulders/elbows (60 kV, 25 mAs), tarsal joints (50 kV, 10 mAs).	Hips (60 kV, 25 mAs), knees (50 kV, 10 mAs).	Hips (65 kV, 25 mAs), knees (50 kV, 15 mAs) left shoulders/elbows (60 kV, 20 mAs), tarsal joints (50 kV, 10 mAs).

**Fig. 5:**
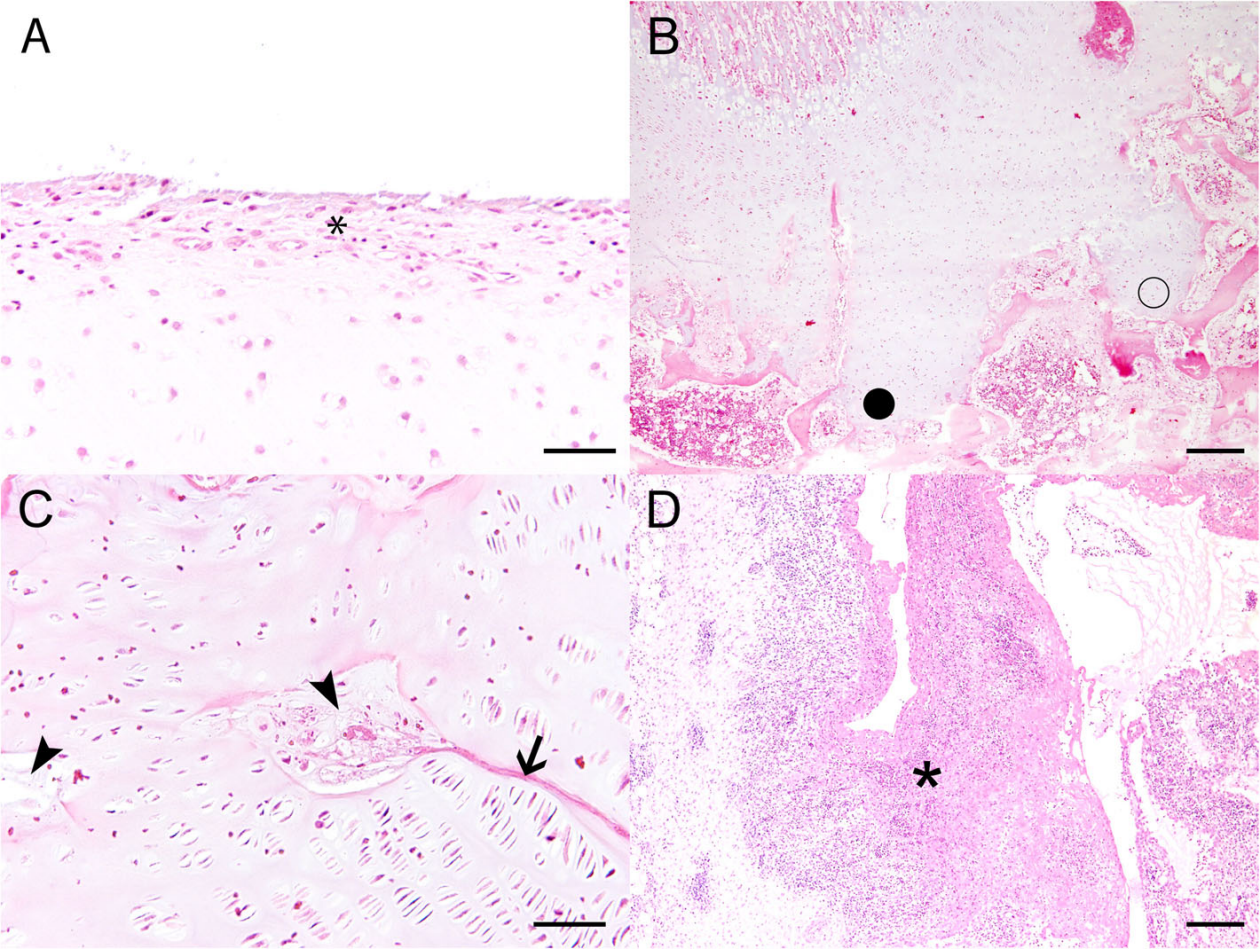
Histologic findings in stifle joints of pig 2. **a** Histopathology revealed a pannus formation at the articular cartilage of the femur with demasking of collagen fibers (asterisk). **b** Within the physis, multifocal cartilage cones (O) were detected. **c** Additional findings in the physis included multifocal chondrocyte degeneration (arrowhead) as well as eosinophilic streaks (arrow). **d** The synovial membrane revealed severe fibrinopurulent (asterisk) inflammation. Hematoxylin and eosin staining, bars = 50 μm (**a**, **c**) and 200 μm (**b**, **d**)

Histologic examinations of the stifle joints were performed at the Department of Pathology of the University of Veterinary Medicine Hannover, Germany. Inflammatory as well as degenerative lesions were found (Fig. 5, Table 4).

**Table 4:** Histologic findings in stifle joints

	Pig 1 (female, 45 kg)	Pig 2 (male, 32 kg)	Pig 3 (female, 39 kg)
**Femur, articular epiphyseal cartilage**		Multifocal pannus formation with demasking of collagen fibers.	
**Femur, physis**	Single cartilage cones.	Multifocal cartilage cones, multifocal chondrocyte degeneration and eosinophilic streaks.	Multifocal cartilage cones, multifocal chondrocyte degeneration, mild medullary fibrosis.
**Stifle joint, synovial membrane**	Mild to moderate, multifocal to coalescent, lympho-plasmahistiocytic synovialitis.	Moderate, fibrinosuppurative, partly lympho-plasma-histiocytic synovialitis.	Mild to moderate, multifocal, lympho-plasma-histiocytic synovialitis.

The femur bones of all three pigs were measured after thorough removal of adjacent tissue. The distal third of the femur of each pig was ashed at the Institute of Animal Nutrition. Serum samples were sent to the Clinic for Swine, Small Ruminants and Forensic Medicine of the University of Veterinary Medicine Hannover to determine the concentration of calcium and phosphorus (Table 5).

**Table 5:** Results of blood analyses and femoral ashing of the three pigs

		Reference values	Pig 1 (female, 45 kg)	Pig 2 (male, 32 kg)	Pig 3 (female, 39 kg)
**Blood analyses**	AP [U/I]	0 - 300^a^	257	175	186
	Ca [mmol/L]	2.5–3.1^a^	2.86	2.53	2.56
	P [mmol/L]	2.8–4.3^a^	2.96	2.69	2.79
	hemoglobin [g/L]	–	0.27	0.4	0.35
**Bone ashing**	Bone length (mm)	–	171	142	158
	Bone diameter (mm)	–	22	19	22
	Total fat-free weight of piece of femur used for analysis (g)	–	27.98	20.80	22.99
	Total ash content of piece of femur used for analysis (g)	–	13.60	9.11	10.92
	DM [g/kg]	–	473	379	422
	Ash [g/kg ffr DM]^d^	493 ± 20.4^b^ 495-524^c^	486	438 ↓	475
	Ca [g/kg ffr DM]^d^	154 ± 8.7^b^ 179-196^c^	180	159	177
	P [g/kg ffr DM]^d^	79.3 ± 3.4^b^ 107-118^c^	83	75.1 ↓	83.5

^a^Reference values for blood in crossbreed grower pigs [35]

^b^Reference values for bone ashing results in this age-group had been previously established for the distal femur epiphysis at the institute [36]

^c^In addition, study findings for the third metacarpal bone of finishing pigs fed different phosphorus sources are recorded [37]. Ranges include results from feeding groups with calculated Ca of 0.78% and P of 0.56% in nursery and Ca of 0.65% and P of 0.43% in fattening diets [37]

^d^Gram per kilogram fat-free dry matter, *AP* alkaline phosphatase

In addition, individual serum samples of the pigs were sent to GD (Gezondheidsdienst voor Dieren, Deventer, the Netherlands) to determine bone markers osteocalcine and C-telopeptid (CTx) reflecting bone metabolism. Serum samples were pooled according to the routine diagnostic procedures. Osteocalcine is indicative of bone formation, while CTx marks bone resorption. While osteocalcine-concentration was reduced (20.0 μg/L [reference value: > 50 μg/L]), CTx was within the reference range (0.17 μg/L [reference value: < 0.2 μg/L]). As a consequence, the osteocalcine:CTx ratio (osteocalcine:CTx = 117.6 [reference range: > 150]) was reduced.

### Interpretation of findings and measures

Diagnostic findings indicated a multifactorial disease pathogenesis.

Clinical findings were typical for both, arthritis caused by *M. hyosynoviae* and osteochondropathia. All pigs were infected by *M. hyosynoviae*. Histologic findings reflected degenerative cartilage and bone alterations characteristic of osteochondrosis (OC). Blood concentrations of calcium and phosphorus gave no evidence of mineral deficiency in the three tested animals.

In a previous experimental study performed at the Institute for Animal Nutrition, University of Veterinary Medicine Hannover, which was involved in this case report, serum *P* values varied between 2.5–2.8 mmol/L in pigs (bw ~ 55 kg) at generous P supply (including inorganic P sources and phytase) but dropped to values close to 1.58 mmol/L at renounced inorganic P and phytase (after 3 weeks of different dietary treatment) [36]. Homeostatic mechanisms control P and Ca concentrations in the blood so that serum concentrations are not considered a reliable indicator of insufficient mineral supply [38]. In general, P serum concentration drops only during severe P deficiency.

The youngest pig 2 showed a reduced mineral content in the femoral bone sample (Table 5). This finding in combination with bone marker values suggested a catabolic status of bone metabolism (bone resorption) and therefore an inadequate mineralization of bones in clinically affected individuals.

Clinical findings of a progressive, shifting lameness, which affects one or more limbs, reluctance to move or to stand up, changing posture of the hind legs were typical of the disease. Moreover, claw lesions were found, which could additionally be a trigger factor for disease due to disturbed body weight balancing, or which could be the result of putting increased weight on the claws due to specific postures.

## Case outcome

In April 2019, in the three different diets used at the early stage of fattening, dietary Ca content was adjusted to 0.7% and total P to 0.48%. The proportion of fermented ingredients in the final liquid diet was restricted to 50%. In parallel, feeding technique was checked and feeding valves were tested and controlled for adequate function on a regular basis.

Between February and May 2019, individual pigs affected by lameness were treated with amoxicillin trihydrate and dexamethasone two-four times at 24 h intervals as previously described. Within these months, the incidence rate decreased to less than 5%.

## Discussion and conclusions

All findings in fatteners with impaired mobility on this farm led to the assumption that *M. hyosynoviae* as an infectious factor was involved in disease pathogenesis in combination with additional factors. A slight increase in synovia volume was found in two of the pigs and in two pigs a subcutaneous edema of the joints was observed. A mild to moderate lymphoplasma-histiocytic inflammation as well as a fibrino-suppurative synovialitis were detected by histology. Infectious arthritis caused by *M. hyosynoviae* often results in decreased profitability for the farmers due to higher medication costs and time-consuming measures that have to be taken, such as segregating diseased pigs in recovery pens. This infectious agent also further impairs skeletal health in fatteners, which is an important welfare issue [2]. In this case, antibiotic treatment with amoxicillin in combination with an anti-inflammatory substance was successful in individual pigs, although mycoplasma species are intrinsically resistant to β-lactam antibiotics [39]. In contrast, MIC values for tiamulin are low for *M. hyosynoviae* (≤0.25 μg/mL) [39], but treatment with this substance was not successful on the farm in the case report. With high probability, the anti-inflammatory parenteral treatment, which was not performed in combination with the in-feed-treatment with a pleuromutilin, was responsible for the improvement. If this was the case, infection with *M. hyosynoviae* might not be the primary cause of the clinical signs, but instead pain either caused by inflammation or by the degenerative joint alterations.

Due to the generally differing outcome of *M.-hyosynoviae*-infection, identifying further influencing factors in affected swine is of high importance. On the farm in question, claw lesions and osteochondropathy were identified as additional factors. During nursery, pigs were kept on plastic flooring, which is characterized by a different hardness compared to concrete flooring in the fattening stable. These differences between both materials in hardness but also surface roughness are risk factors for the development of claw lesions as a consequence of mismatching in horn quality and underground [40]. Piglets experience a sudden change in underground after moving to the fattening unit without an adaptation period. The observed claw lesions were indicative of abrasive injuries (sole erosions), but can also be the consequence of extended resting periods due to painfulness while moving (skin lesions at the coronary band region). In general, additional factors can support the development of claw lesions, e.g., genetically determined asymmetries in inner and outer claws and abnormal toe angles, but also nutrient deficiency, e.g., biotin [41, 42], which were not suspected in the present case. Claw lesions can be the consequence of degenerative joint diseases but also a risk factor for the development thereof such as OC [43]. Any kind of cartilage pre-damage might contribute to the adhesion of *M. hyosynoviae* [44]. A negative impact of P deficiency - suspected here at least in one animal - on the function of the connective tissue has been shown in other species [45]. Hypothetical low intake of P would include low P concentrations in the blood as acute P deficiency, which was only observed in one pig (Table 5). The determined bone markers hinted at a stimulated bone resorption maybe as a consequence of marginal P supply of individuals with higher daily weight gain [46, 47].

Nevertheless, the hypothesis of an additional nutritional impact on disease development could only be supported by bone composition in one pig in this case. The comparably low bone mineral content in combination with the bone marker result might suggest that bone formation with respect to mineral accretion was reduced. Bone ashing in pigs is a further diagnostic approach to verify the suspicion of impaired mineralization. Standardization of the method has been improved in recent years so that preliminary reference values could be elaborated for the femur [36]. Bone ash diagnostic was found to be appropriate for diagnostic evaluation of marginal supply with minerals lasting at least 3 weeks [36]. In growth periods with insufficient Ca and P supply, at first, the total bone mass is reduced, while bone formation and composition might be maintained. A lower ratio of the diameter of the long bones to body mass can be the consequence [36]. The pressure on the end of bones covered by cartilage depends on body mass and the area of contact within the joint. It could be that also in young pigs – as observed in growing dogs- the dietary P supply affects the strength of muscles that keep the bones in the right position [45]. With an insufficient P intake and impaired muscle tonus, there is a predisposing effect for alterations of the cartilage especially in pigs with high growth rates.

Analysis of diet composition and feeding anamnesis are fundamental in the diagnostic procedure. The authors assume that a marginal mineral supply during a specific juvenile phase of life with high growth rates might be predisposing factors of disease development in later life. The three pigs examined in this study were slightly heavier (approximately 3 kg) than the average pigs in the respective age-group, so that high daily weight gain can be assumed. In individuals with high growth rates (~ 900 g average daily weight gain), the uptake of digestible P might have been insufficient at the end of nursery/beginning of fattening, especially when the phytase content in the final diet is reduced by a relatively high proportion of phytase-free fermented ingredients. To diagnose a marginal mineral supply in critical growth phases, not only the demand of the pigs with respect to feed intake and growth rate, but also for bone mineralization should be considered.

A ratio of digestible Ca to digestible P for adequate mineralization of bones and optimal growth performance was found to be approximately 1.23:1 in cases when digestible P met the requirements [48]. Excess Ca in combination with P concentrations below recommended requirements led to decreased growth rates [48]. Both requirements were fulfilled in this case report with low P concentrations and a relatively wide Ca:P ratio in the final diet. Estimates for Ca requirements of Ca in growing pigs range from 6.3–4.2 g/kg DM in pigs with 20–80 kg bw [19, 20]. Different batches of compound feed vary in P and Ca concentrations, which can lead to deficiencies in short periods depending on batch size. Labeled diet compositions are only based on an analysis in the first charge. In general, digestibility of dietary P is markedly improved by fermenting the liquid diet before offering it to the animals and by adding phytase to the compound feed [49–51]. Both strategies were applied on this farm.

In the three examined pigs in this study, histologic findings were only indicative of osteochondropathy and not mineral or vitamin D deficiency, which would be characterized by distinct morphologic entities as e.g. failure of newly formed osteoid to mineralize [52, 53]. As metabolic bone diseases are usually reversible, any typical histologic changes might had been present at an earlier point in time and would most likely have been superimposed by the degenerative processes at the time of examination. Some studies support the multifactorial pathogenesis of osteochondropathy but there is lack of evidence for an interaction with mineral deficiency [54, 55]. Under experimental conditions, hypophosphataemia was found to cause focal cartilage lesions, which were different from cartilage degeneration typical of osteochondrosis [56].

At the beginning of being involved in this clinical case, the primary hypothesis was that the development of the disease was a multifactorial process starting with an impaired bone mineralization and triggering the development of osteochondropathy of the joints. Pigs with high growth rates develop high pressure on cartilage surfaces of the long bones. In the case that muscular forces are weakened due to a marginal P supply, deviations in limb axes with the consequence of improper biomechanical stress on joint cartilage surfaces, aseptic inflammation and subsequent pain during movement might occur. Whether cartilage alterations as shown in these pigs are predisposing factors for colonization and infection with *M. hyosynoviae* remains hypothetical. Some authors have considered that OC could be a predisposing factor for joints to be infected with *M. hyosynoviae* [44]. However, these assumptions were refuted by authors who did not find *M. hyosynoviae* any more frequently in individuals at the slaughterhouse with OC than in individuals without OC [3].

So far, the potential triad involved in disease development can be covered by the most straightforward diagnostic steps reported in veterinary practice; namely, i) comparison of recommended dietary P levels with those analyzed in the investigated case, during specific growth phases with expected high growth rates based on available farm-specific production and feed data, ii) bacteriological diagnostic of articular samples and iii) histologic examination of articular tissue. The new blood markers for diagnosis of mineral deficiency should be validated in the context of more practical cases, because they might be a promising new diagnostic tool for assessing skeletal health. Bone marker determination should be recommended especially in pigs with high daily weight gains showing stiff walking, tripping and reluctancy to stand up, in order to assess the relation between bone catabolism and bone mineralization. In addition, we recommend in cases of sudden increased lameness to look at recent findings without neglecting the period before the episodes, including the previous dietary supply with P, especially of individuals with higher daily weight gains.

Finally, the question concerning the degree of impact of either P deficiency or osteochondropathy as well as claw lesions on the disease and especially on disease pathogenesis in *M. hyosynoviae* infection cannot be answered in this case. Thus, experimental studies under standardized conditions, also taking various genetic backgrounds into account, would be necessary.

## Data Availability

Data sharing is not applicable to this article as no datasets were generated or analyzed during the current study.
